# Relationship between sarcopenia and orthostatic blood pressure recovery in older falls clinic attendees

**DOI:** 10.1007/s41999-023-00775-0

**Published:** 2023-04-08

**Authors:** Eoin Duggan, Silvin P. Knight, Roman Romero-Ortuno

**Affiliations:** 1 Discipline of Medical Gerontology, School of Medicine, Trinity College Dublin, Dublin, Ireland; 2 Falls and Syncope Unit (FASU), Mercer’s Institute for Successful Ageing, St James’s Hospital, Dublin 8, Ireland

**Keywords:** Sarcopenia, Blood pressure recovery, Orthostatic hypotension, Skeletal muscle pump, Orthostasis, Older people

## Abstract

**Purpose:**

Sarcopenia and delayed orthostatic blood pressure (BP) recovery are two disorders increasingly associated with adverse clinical outcomes in older adults. There may exist a pathophysiological link between the two via the skeletal muscle pump of the lower limbs. Previously in a large population-based study, we found an association between probable sarcopenia and orthostatic BP recovery. Here, we sought to determine the association between confirmed sarcopenia and orthostatic BP recovery in falls clinic attendees aged 50 years or over.

**Methods:**

One hundred and nine recruited patients (mean age 70 years, 58% women) underwent an active stand with non-invasive beat-to-beat haemodynamic monitoring. Hand grip strength and five-chair stands time were measured, and bioelectrical impedance analysis was performed. They were then classified as robust, probable sarcopenic or sarcopenic as per the European Working Group on Sarcopenia in Older People guidelines. Mixed effects models with linear splines were used to model the effect of sarcopenia status on orthostatic BP recovery, whilst controlling for potential confounders.

**Results:**

Probable sarcopenia was identified in 32% of the sample and sarcopenia in 15%. Both probable and confirmed sarcopenia were independently associated with an attenuated rate of recovery of both systolic and diastolic BP in the 10–20 s period after standing. Attenuation was larger for confirmed than probable sarcopenia (systolic BP β − 0.85 and − 0.59, respectively, *P* < 0.01; diastolic BP β − 0.65, − 0.45, *P* < 0.001).

**Conclusion:**

Sarcopenia was independently associated with slower BP recovery during the early post-stand period. The potentially modifiable effect of the skeletal muscle pump in orthostatic haemodynamics requires further study.

## Key Summary Points

**Aim:**

To determine the association between sarcopenia and orthostatic blood pressure (BP) recovery in falls clinic attendees aged 50 years or older.

**Findings:**

Sarcopenia had a significantly slower rate of BP recovery (both systolic and diastolic) during the 10–20 s period after standing, independent of confounders.

**Message:**

Sarcopenia had a modest but significant attenuating effect on early orthostatic haemodynamics.

## Introduction

Sarcopenia is emerging as one of the major age-associated health challenges of the twenty-first century [1]. Accumulating evidence suggests that sarcopenia is one of the central pathological processes driving physical frailty [2] and it is implicated in a wide range of adverse health outcomes in older adults [3, 4]. Additionally, sarcopenia is associated with cardiovascular diseases including coronary artery disease, atrial fibrillation, and heart failure [5, 6].

Delayed blood pressure (BP) recovery on standing, previously under-recognised in comparison to the classic syndrome of orthostatic hypotension (OH), is also emerging as a key driver of falls and adverse clinical outcomes in older adults [7, 8, 9]. Whereas previously OH was defined in terms of a sustained drop in BP (≥ 20 mmHg systolic and/or ≥ 10 mmHg diastolic) at 3 min after standing, the prevalence of delayed BP recovery has been identified through the use of beat-to-beat orthostatic BP measurement in a number of population cohort studies [10, 11].

There is reason to suspect a pathophysiological link between sarcopenia and both delayed BP recovery and OH, given the role of the skeletal muscle pump in BP recovery and maintenance. The skeletal muscle pump is thought to aid venous return to the heart during exercise and standing, via rhythmic activity of the muscles of the lower limbs [12]. Loss of muscle strength and mass in sarcopenia could affect skeletal muscle pump function, leading to slower BP recovery and OH. This could then lead to falls, fractures, increased morbidity and even subsequent mortality. The clinical importance here is that unlike many other causes of OH, sarcopenia is potentially preventable and/or reversible through physical exercise and nutrition interventions [13].

Two previous studies found significant association between sarcopenia and OH measured with intermittent oscillometric devices [14, 15]; however, the relationship between sarcopenia and beat-to-beat orthostatic BP recovery is less well known. Our previous research in a large population-based cohort found an association between probable sarcopenia and orthostatic BP recovery and OH at 30 s [16]; however, we were limited to measures of probable sarcopenia in that cohort. The present study sought to determine the association between bioelectrical impedance analysis-confirmed sarcopenia and continuous non-invasive orthostatic BP recovery in falls clinic attendees aged 50 years or over.

## Methods

Attendees to the Falls and Syncope Unit (FASU) at St. James’s Hospital, Dublin, Ireland were recruited prospectively between November 2021 and November 2022. The FASU setting has been described elsewhere [17]. Inclusion criteria for the study were: age 50 years or older, able to provide written informed consent, able to mobilise independently (with or without aid) and able to transfer with minimal assistance of one person from lying to standing. Exclusion criteria were: history of confirmed or presumed neurogenic OH, or contraindication to bioelectrical impedance analysis (e.g. presence of an indwelling electronic device such as a cardiac pacemaker). Ethics approval for the study was granted from the Tallaght University Hospital/St. James’s Hospital Joint Research Ethics Committee (Project ID: 0221; approval date: 4 May 2021). Approval was also granted by St James’s Hospital Research & Innovation Office (Reference: 6567, approval date: 26 July 2021). All included patients provided written informed consent to partake in the study. The study adhered to the World Medical Association Declaration of Helsinki on ethical principles for medical research involving human subjects.

Participants’ disease status and medication use were obtained from a combination of self-report, medical chart review and correspondence from the participants’ General Practitioner where available. Multimorbidity was defined as two or more chronic conditions and polypharmacy as five or more regular medications excluding supplements. Physical frailty status was determined using the SHARE Frailty Instrument for Primary Care (SHARE-FI) [18]. Cardiovascular medication use was coded as ‘Yes’ if the participant was taking one or more of the following medications (coded using the Anatomical Therapeutic Chemical Classification (ATC)): anti-arrhythmics (ATC C01), anti-hypertensives (ATC C02), diuretics (ATC C03), vasodilators (ATC C04), beta-blocking agents (ATC C07), calcium channel blockers (ATC C08), or agents acting on the renin–angiotensin system (ATC C09). Psychotropic medication use was coded as ‘Yes’ if the participant was taking one or more of the following: anti-epileptics (ATC N03A), anti-psychotics, anxiolytics, hypnotics or sedatives (ATC N05), or anti-depressants (ATC N06A).

Hand grip strength (HGS) was assessed using a Jamar Hydraulic Hand Dynamometer (Performance Health, Wisconsin, USA). The maximum value of two consecutive measurements taken on the left and right hands, whilst seated, was used. Values were rounded to the nearest 2 kg, as per the precision of the device. The time taken on the five-chair stands test (5CST) was measured as the time to the nearest centi-second taken for a participant to stand up and sit back down again, as fast as possible, five times from a standard chair (approximate seat height 43 cm). Height was measured to the nearest 0.01 m (Seca 222 Stadiometer, Seca Ltd, Birmingham, UK). Bioelectrical impedance analysis was performed with the TANITA^®^ DC-430 MAP Body Composition Analyser (Tanita Europe, Amsterdam, The Netherlands). Participants stood barefoot on the scale, which also provided weight measurement to the nearest 0.01 kg. Participants were asked to remove their outerwear and empty their pockets, and 0.5 kg was entered as a standard tare value for clothing. Appendicular skeletal muscle mass was calculated using the Sergi equation [19]. The European Working Group on Sarcopenia in Older People (EWGSOP) revised cutoffs for probable sarcopenia (HGS of less than 27 kg in men and 16 kg in women and/or 5CST greater than 15 s) and sarcopenia (muscle mass less than 20 kg in men and 15 kg in women) were used [20].

Participants underwent a continuously monitored active stand test as per recommended guidelines [21], with a signal calibration and resting phase of 5–10 min, followed by standing as quickly as possible (with assistance if necessary) and remaining standing quietly for 3 min. Continuous non-invasive beat-to-beat BP was measured with a Finapres^®^ Nova (Finapres Medical Systems, Amsterdam, The Netherlands). The signals were calibrated using brachial artery measurements and Physiocal™, with the height correction unit correcting for hydrostatic pressure differences.

Beat-to-beat signals were exported from the Finapres^®^ Nova software and analysed using custom written software in MATLAB^®^ version 9.11 (The MathWorks, Inc., Natick, MA, USA). Signals were inspected for poor quality and those with excess noise or artefacts were excluded. If there was a suspicion of delay between the marker for standing and actual standing, the height correction unit signal was examined and the stand time corrected. The baseline signal was taken as the mean from 60 to 30 s before standing. Ten-second averages were taken from 0 to 180 s after standing and used in the analysis. OH at 30 s (OH30) was defined as a drop of SBP ≥ 20 mmHg and/or DBP ≥ 10 mmHg from baseline values.

Statistical analysis was performed with Stata^®^ version 15.1 (StataCorp LLC, College Station, TX, USA). The distributions of continuous variables were examined visually with histograms and distributional diagnostic plots and tested using the skewness kurtosis test for normality [22]. For the demographics of the sample, continuous normally distributed variables were summarised by the mean and standard deviation (SD), and non-normal continuous variables by the median and interquartile range (IQR). Proportions were given as count and percentage. Given that sarcopenia status was a three-level ordinal variable (i.e. robust, probable sarcopenia and sarcopenia), trends in continuous variables across those three levels were examined with Kendall’s rank correlation coefficient [23, 24], whilst trends in dichotomous variables were examined using Chi-square for trend. Additionally, the effect of sarcopenia status on systolic and diastolic BP after standing was represented graphically, from standing to 180 s, with the mean change in BP and standard error of the mean, by sarcopenia group.

Given that multiple repeated measures of BP were taken for each participant, a statistical method that accounted for this and allowed adjustment for potentially confounding factors was needed. As per previous work by our group and others, we implemented mixed-effects models with piecewise linear splines to model time points 0–10, 10–20, 20–30, 30–40 and 40–180 s after standing [16, 25, 26]. Residual variance was modelled with a first order autoregressive process to account for the strong correlation between adjacent time points. The linear splines were entered into the model as independent parameters along with interaction terms with sarcopenia status (robust—0; probable sarcopenia—1; sarcopenia—2) and potential confounders as covariates. The potential confounders considered were: age, sex, diabetes, hypertension, cardiovascular medications and psychotropic medications. In addition, in an attempt to isolate the effect of the skeletal muscle pump’s contribution to BP response from that of the main cardiac pump, we also controlled for heart rate (HR), as an interaction term with each linear spline. Variables that theoretically lay on the causative pathway to sarcopenia, for example multimorbidity [27], were considered mediators rather than confounders and as such not controlled for in the models. Fully adjusted beta (β) coefficients with 95% confidence intervals (CI) for the five time periods were obtained from the mixed-effects models outputs. The level of statistical significance was defined as *P* < 0.05 throughout.

## Results

Over the recruitment period, 123 patients consented to participate in the study. Of these, 5 had a contraindication to or declined BIA, 7 had probable or confirmed neurogenic OH, and in 2 there was no active stand test or the active stand signal was of poor quality. Thus, the included sample size was 109. Overall, the mean age of the included sample was 70 years (SD 10.5, range 5093), and 58% were women.

The descriptives of the included sample grouped by sarcopenia status are presented in Table 1. Statistically significant trends with sarcopenia status were identified for increasing age and presence of multimorbidity, physical frailty, polypharmacy, psychotropic medications, and increasing 5CST. Weight, height, HGS and appendicular skeletal muscle mass significantly decreased as sarcopenia status increased. The difference in the prevalence of OH30 across sarcopenia status did not meet statistical significance.

**Table 1:** Characteristics of the sample grouped by sarcopenia status

	Robust (58/53.2%)	Probable sarcopenia (35/32.1%)	Sarcopenia (16/14.7%)	*P*
Age (years) (mean/SD)	66.1 (9.8)	73.8 (8.8)	76.0 (11.2)	< 0.001
Female (n/%)	30 (51.7)	24 (68.6)	9 (56.3)	0.383
Weight (kg) (median/IQR)	75.4 (18.7)	73.8 (19.4)	57.7 (16.8)	0.034
Height (cm) (mean/SD)	169.3 (9.5)	165.3 (7.7)	162.9 (9.1)	0.007
BMI (kg/m^2^) (median/IQR)	25.5 (4.8)	27.8 (4.3)	23.0 (5.8)	0.502
5CST (s) (median/IQR)	12.0 (2.8)	19.7 (6.2)	18.7 (11.1)	< 0.001
HGS (kg) (median/IQR)	29.0 (14.0)	20.0 (11.0)	16.5 (9.0)	< 0.001
ASMM (kg) (median/IQR)	19.2 (6.9)	18.6 (6.5)	14.5 (6.3)	0.034
Frail (n/%)	1 (1.7)	10 (28.6)	6 (37.5)	< 0.001
Multimorbidity (n/%)	41 (70.7)	31 (88.6)	15 (93.8)	0.014
Polypharmacy (n/%)	21 (36.2)	21 (60.0)	11 (68.8)	0.006
Hypertension (n/%)	26 (44.8)	18 (51.4)	9 (56.3)	0.368
Diabetes (n/%)	8 (13.8)	7 (20.0)	1 (6.3)	0.756
Cardiovascular Meds (n/%)	29 (50.0)	16 (45.7)	10 (62.5)	0.564
Psychotropic Meds (n/%)	11 (19.0)	18 (51.4)	7 (43.8)	0.006
OH30 (n/%)	14 (24.1)	13 (37.1)	7 (43.8)	0.083

BMI, body mass index; 5CST, five-chair stands test; HGS, hand grip strength; ASMM, appendicular skeletal muscle mass; OH30, orthostatic hypotension at 30 s after standing; Meds, medications; SD, standard deviation; n, number; IQR, interquartile range

**Fig. 1:**
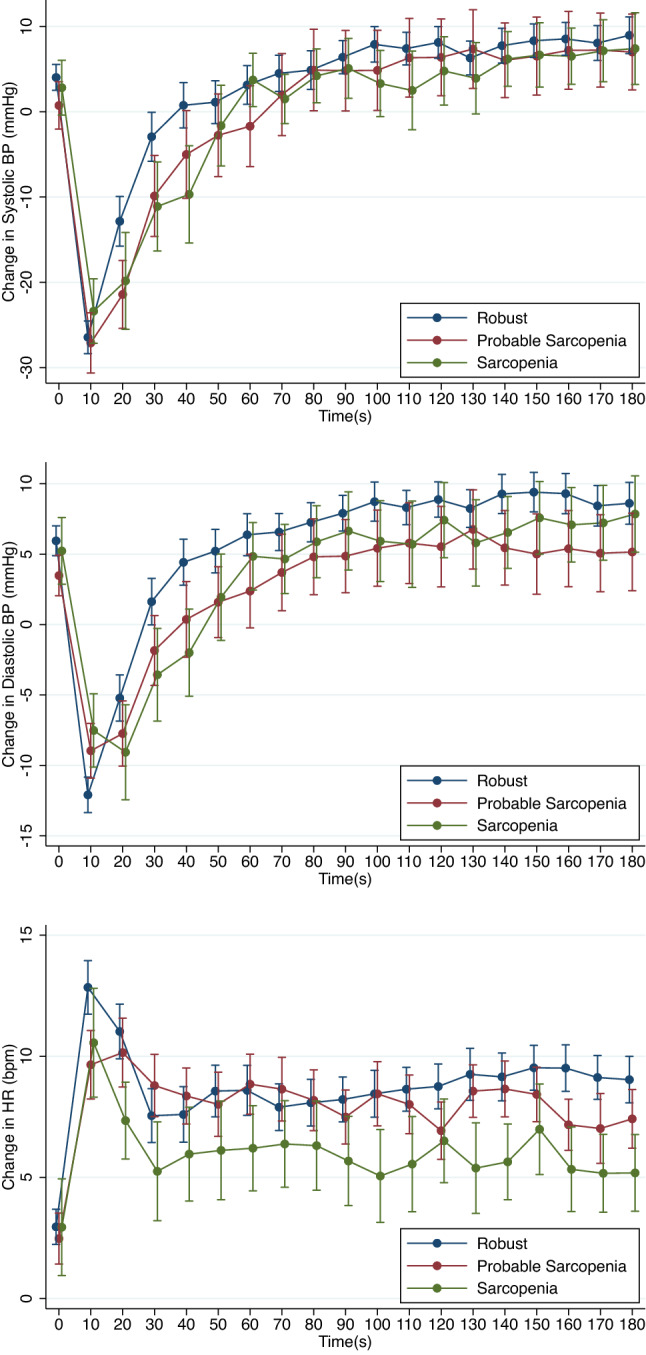
Mean change in systolic blood pressure, diastolic blood pressure (mmHg) and heart rate (beats per minute—bpm) after standing grouped by sarcopenia status. Unadjusted. Error bars: standard error of the mean

The raw data-based mean changes in systolic BP, diastolic BP and HR after standing, grouped by sarcopenia status, are displayed in Fig. 1. Visually, the probable sarcopenia and sarcopenia groups appeared to have slower recoveries from the BP nadir, and there seemed to be a less pronounced HR response especially in the sarcopenia group.

**Fig. 2:**
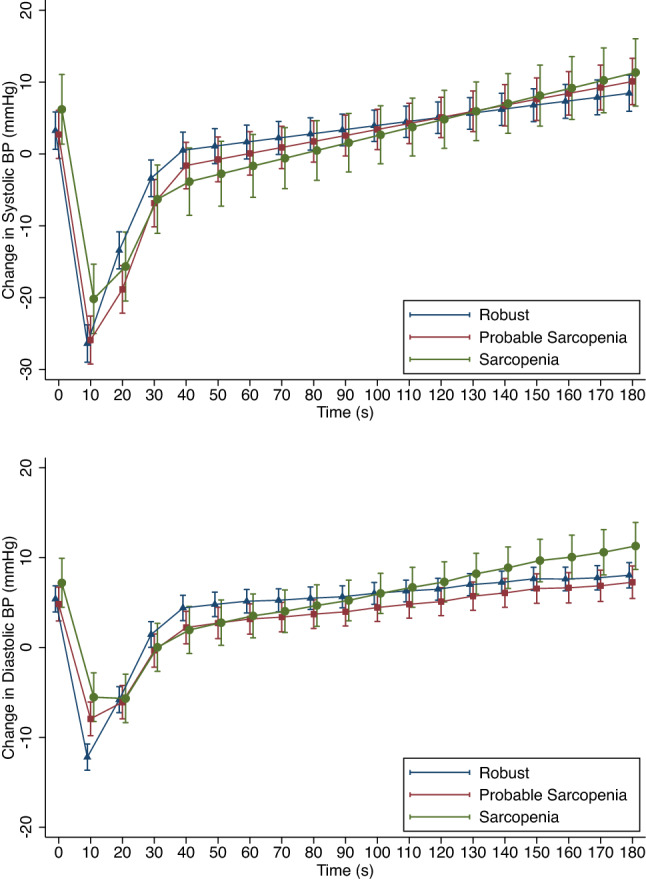
Predicted means and standard error of the mean from mixed-effects models for change in systolic and diastolic blood pressure (mmHg) after standing, grouped by sarcopenia status. Models were adjusted for age, sex, diabetes, hypertension, cardiovascular and psychotropic medications and heart rate

The β coefficients and CI for the five time points in the mixed-effects models for systolic and diastolic BP, by sarcopenia status, controlling for confounders, are presented in Table 2. To aid interpretation, the predicted marginal means for change from baseline in systolic and diastolic BP, grouped by sarcopenia status, are shown in Fig. 2. There was significantly attenuated recovery of both systolic and diastolic BP during the 10–20 s period post-standing for both the probable sarcopenia and sarcopenia groups when compared to the robust group, as evidenced by the negative coefficients during a period where the overall BP trend was positive. However, for both systolic and diastolic BP, the sarcopenia group had a more attenuated recovery than the probable sarcopenia group. The probable sarcopenia and sarcopenia groups also had an attenuation in the initial drop in BP on standing as evidenced by the significant positive β coefficients at a time when the overall BP trend was negative.

**Table 2:** Beta-coefficients and 95% confidence intervals for the effect of sarcopenia status on the change in systolic and diastolic blood pressure (mmHg) from baseline at time intervals 0–10 s, 10–20 s, 20–30 s, 30–40 s and 40–180 s after standing

	0–10 s β (95% CI)	10–20 s β (95% CI)	20–30 s β (95% CI)	30–40 s β (95% CI)	40–180 s β (95% CI)
*Systolic BP*					
Probable sarcopenia	0.10 (− 0.28, 0.48)	− 0.59 (− 0.97, − 0.21)**	0.20 (− 0.18, 0.58)	0.13 (− 0.24, 0.51)	0.03 (− 0.03, 0.08)
Sarcopenia	0.32 (− 0.18, 0.83)	− 0.85 (− 1.35, − 0.34)**	− 0.07 (− 0.57, 0.44)	− 0.15 (− 0.64, 0.35)	0.05 (− 0.02, 0.13)
*Diastolic BP*					
Probable sarcopenia	0.48 (0.26, 0.71)***	− 0.45 (− 0.68, − 0.23)***	− 0.15 (− 0.37, 0.07)	− 0.04 (− 0.26, 0.18)	0.01 (− 0.02, 0.04)
Sarcopenia	0.49 (0.20, 0.78)**	− 0.65 (− 0.95, − 0.36)***	− 0.16 (− 0.45, 0.14)	− 0.10 (− 0.39, 0.19)	0.04 (< − 0.01, 0.08)

Models were adjusted for age, sex, diabetes, hypertension, cardiovascular and psychotropic medications and heart rate. *P < 0.05, **P < 0.01, ***P < 0.001

## Discussion

In this study, we determined the association between sarcopenia (probable and bioimpedance-confirmed) and continuous orthostatic BP recovery in 109 falls clinic attendees aged 50 years or older. We observed that both probable sarcopenia and sarcopenia were associated with an attenuated rate of recovery of both systolic and diastolic BP in the 10–20 s period after standing, with a larger attenuation for the sarcopenia group compared to the probable sarcopenia group. These effects were independent of age, sex, diabetes, hypertension, cardiovascular and psychotropic medications, and heart rate response, supporting the hypothesis that they could reflect the effect of sarcopenia on the skeletal muscle pump of the lower limbs in supporting early post-standing haemodynamic stabilisation. The results are strengthened by the comprehensive characterisation of the sample, allowing robust adjustment for potential confounders, the continuous non-invasive beat-to-beat BP measurements, and the use of the consensus EWGSOP revised definitions for the diagnosis of sarcopenia.

These results extend our previous population-based work on probable sarcopenia [16] and, to the best of our knowledge, show for the first time an association between confirmed sarcopenia and delayed BP recovery using continuous non-invasive orthostatic haemodynamic measurements. Findings are clinically important as even in the absence of OH at 30 s, delayed BP recovery in the early post-standing phase can contribute to reduced cerebral perfusion and increase the risk of falls and syncope [8, 28, 29], which in turn can lead to fractures, hospital admissions, loss of functional independence, and risk of institutionalisation [9, 30]. Although sarcopenia can directly lead to falls [31], our results suggest the existence of an indirect mechanism whereby sarcopenia could also contribute to falls by impairing early post-standing BP recovery. The clinical importance of this is that unlike many other causes of OH, sarcopenia is a potentially reversible risk factor. Recent evidence from the SPRINTT trial found that a multicomponent intervention could reduce sarcopenia and mobility disability in older adults [32]. If similar interventions were implemented in those at risk of OH via the sarcopenia-mediated impaired orthostatic BP recovery pathway, lower limb strengthening and nutrition could potentially reduce the risk of subsequent falls by optimising the skeletal muscle pump and BP recovery.

This study adds to the importance of identifying and treating sarcopenia. The recent World Guidelines for Falls Prevention and Management for Older Adults [33] advise muscle strength measurement as part of a multifactorial falls assessment but they do not give any specific recommendations regarding sarcopenia diagnosis or treatment. Given the potential existence of a second OH-mediated pathway by which sarcopenia could lead to falls, further research may be warranted as to the role of sarcopenia identification in falls risk assessment clinics.

The proposed mechanism by which sarcopenia is linked to OH is via the skeletal muscle pump. Based on our results, we hypothesise that sarcopenia may lead to reduced activity of the skeletal muscle pump during early orthostasis, and hence reduced venous return from the lower limbs resulting in a slower or incomplete recovery of BP. However, despite the theory of its existence being nearly 100 years old [34], the skeletal muscle pump remains understudied and its precise contribution to orthostatic haemodynamics in older adults is uncertain [35]. Some studies have examined the role of the skeletal muscle pump in specific populations. Stewart and colleagues [36] investigated muscle pump function in patients with postural orthostatic tachycardia syndrome finding that the amount of blood ejected by the skeletal muscle pump is related to calf circumference and calf blood flow whilst supine. Kondo et al. [37] showed that in patients with heart failure with reduced ejection fraction, the skeletal muscle pump indirectly assists cardiac output by increasing venous return.

To the best of our knowledge, no studies had assessed the function of the skeletal muscle pump in sarcopenia, although a number have examined the relationship between sarcopenia and OH. A higher prevalence of oscillometric-determined OH in older participants with sarcopenia has been found when measured lying and standing [14, 38], and using a head-up tilt test [15]. Other studies have indirectly looked at muscle strength or mass separately. For instance, diastolic BP recovery, as measured with beat-to-beat BP, in the 15–30 s and 30–60 s periods post-standing was associated with worse 5CST performance [28]. De Bruine et al. [39] found that participants with the worst performance on 5CST, compared to intermediate and best groups, had significantly higher odds of OH measured with beat-to-beat BP. Chen et al. [40] found significantly increased odds of OH with auscultatory BP measurements for those with weak grip strength, whilst Roca et al. [41] reported similar results with oscillometric measurement of BP. Looking at muscle mass alone, Benton et al. [42] found greater drops in BP measured oscillometrically on standing, in a low muscle mass group compared to a normal muscle mass group, both before and after overnight fast.

Thus, the results of these previous studies suggest first a relationship between sarcopenia and OH, and second the existence of a relationship between skeletal muscle pump function and BP. By demonstrating that sarcopenia is related to the continuous BP recovery after standing, independent of heart rate, our study supports these previous findings and gets closer to investigating the mechanism at play. It is clear, however, that further research is needed to characterise the skeletal muscle pump.

Additionally, in our study, we found an attenuated fall in diastolic BP in the initial 10 s after standing for the probable sarcopenia and sarcopenia groups compared to the robust group, as demonstrated by the significant positive β coefficients during a time when the overall BP was falling. It has been argued that the initial drop in BP on active standing, which is not seen in passive changes in posture, i.e. the head-up tilt [43], may be more physiological than pathological and mostly related to the effort and speed of standing [44, 45]. As applied to our results, this is plausible as those with sarcopenia would be expected to have a lower standing speed with a slower initial fall in BP and a less pronounced nadir.

The present study did not replicate a finding of our previous population-based study, namely the statistical significance of the association between probable sarcopenia and OH30 [16]. This may have been due to the imprecision of the estimates, given the small sample size in the present study and the higher variability in the data as evidenced by the wider standard errors shown in the data visualisations. This suggests that the magnitude of the muscle pump effect is at best modest, and this is in keeping with known physiology. Indeed, the predominant mechanisms known to be involved in BP recovery after standing are the baroreflex-mediated splanchnic and peripheral vasoconstriction and the increased cardiac output via vagal withdrawal causing an increase in heart rate [46]. Yet, even if the muscle pump contribution is modest, it is one that is potentially more modifiable, via physical training to improve muscle mass and strength, than other autonomic nervous system mechanisms.

As well as the relatively small sample size, limitations of the present study include the known limitations of bioimpedance analysis for determination of muscle mass; for example, measurements can be influenced by hydration status and the measurements themselves are an estimate referenced to a dual-energy X-ray absorptiometry standard, and do not represent the gold standard [47]. Patients were not required to fast for the minimum required time of 4–6 h before measurements. Furthermore, although we collected medication records, no information was available in relation to timing of medication ingestion in relation to the active stand test routinely performed in the clinic. Different medications will have different half-lives, and therefore their effect on the BP response after standing may vary depending on the time of ingestion in relation to the active stand test.

In conclusion, in this study, we found that older falls clinic patients with sarcopenia had a slower rate of recovery of BP early after standing, as measured by continuous non-invasive haemodynamics. This may put them at risk of OH, falls and fractures. This has important implications for the role of sarcopenia identification in falls risk assessment. The more detailed quantification and potentially modifiable effect of the skeletal muscle pump in orthostatic haemodynamics requires further study.

## Data Availability

The data that support the findings of this study are not openly available due to the conditions of ethics approval for the study and current data protection legislation. Dependent on compliance with data protection legislation and ethical approval they may be available from the corresponding author upon reasonable request.
